# Supplementary material for: Measuring mRNA translation in neuronal processes and somata by tRNA-FRET
Source: Nucleic Acids Res. 2020 Jan 24;48(6):e32. doi: 10.1093/nar/gkaa042 (PMC7102941; doi:10.1093/nar/gkaa042)

Figure 1– figure supplement 1. tRNA isoacceptor distribution is similar pre- and post-labeling.

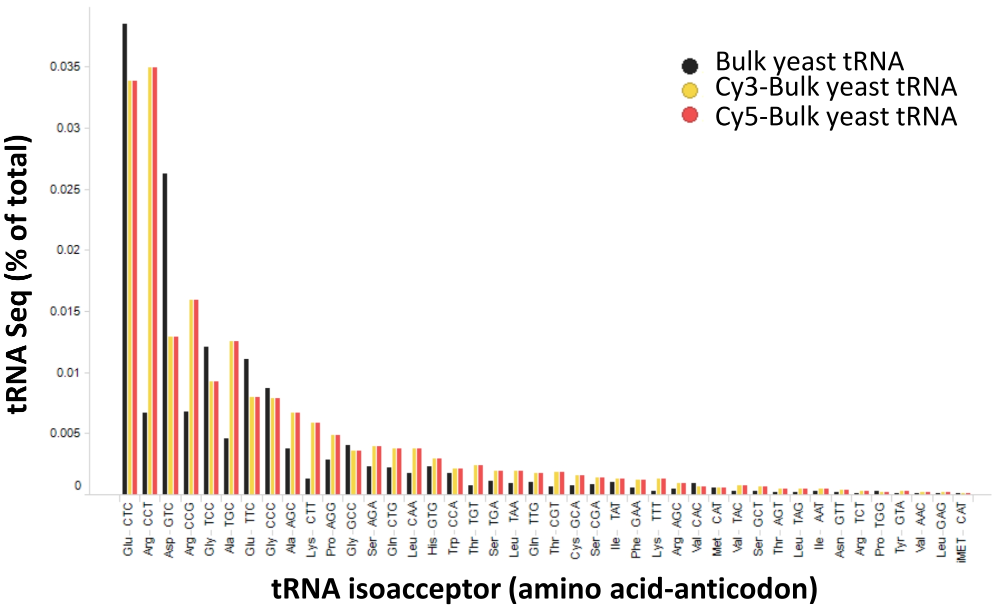

Figure 1– figure supplement 2. Fluorescently-labeled tRNA is translationally functional in HeLa cells.

A.

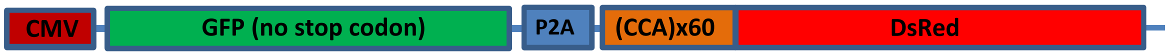

B.

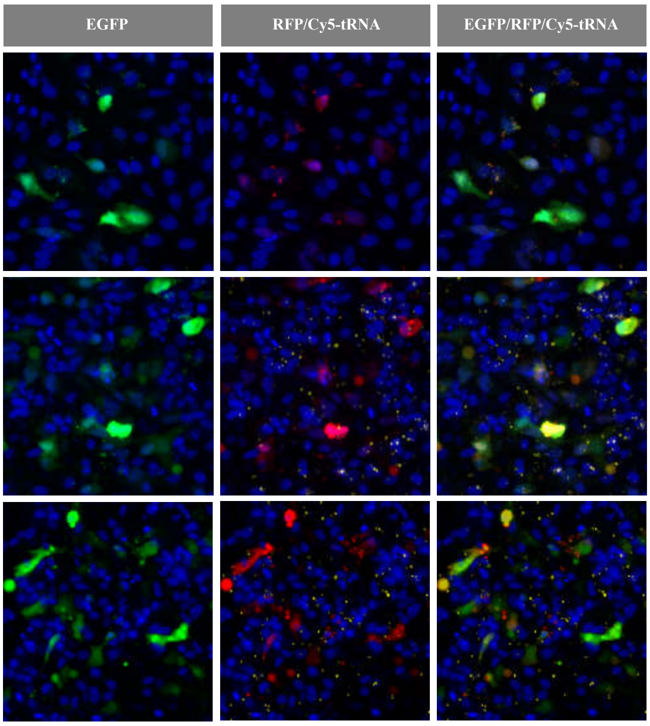

C.

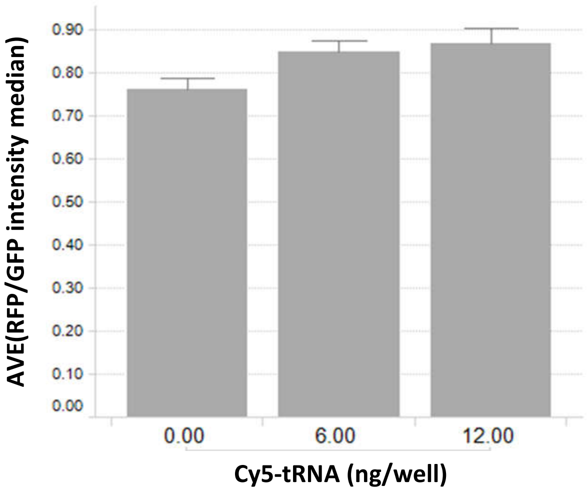

Figure 1– figure supplement 3. Levels of peIF2α return to baseline 48h following application of tRNA

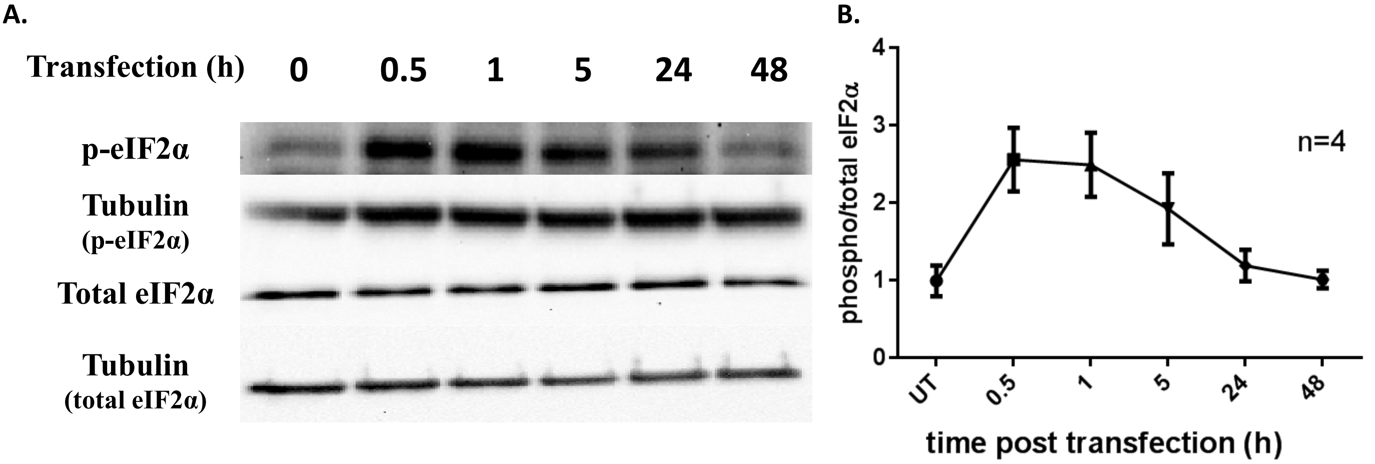

Figure 1– figure supplement 4. tRNA co-localization levels with Calreticulin are higher in glial cells than in neurons

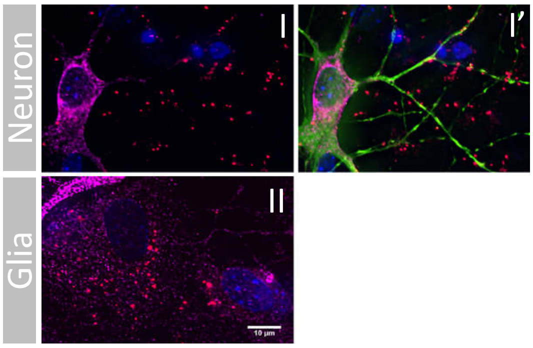

Figure 1 – figure supplement 5. Mature culture has more tRNA puncta in the soma and more condensed tRNA puncta regions ("hot spots") in comparison to young culture.

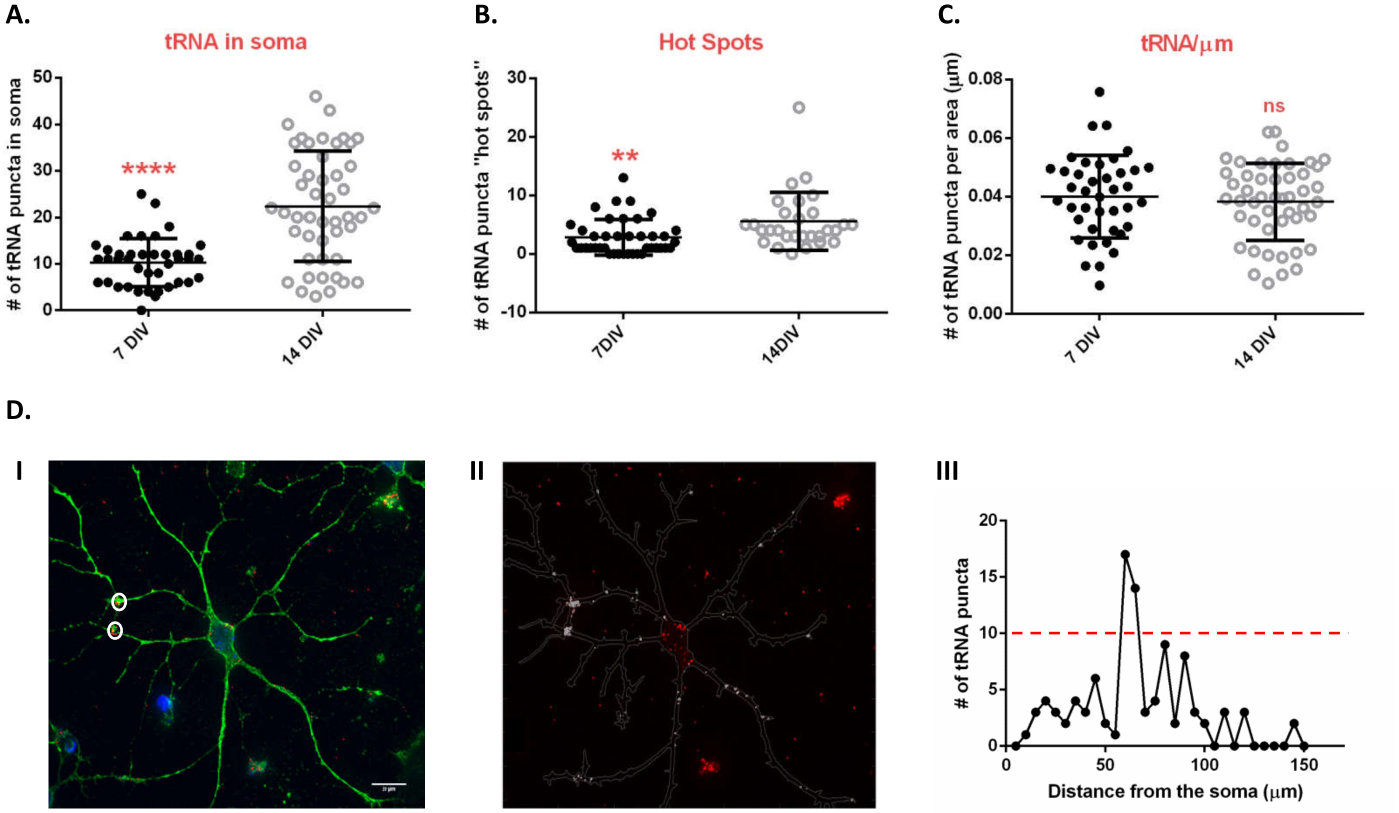

Figure 2 – figure supplement 1. Total amount of tRNA puncta remains unchanged in all measured time points

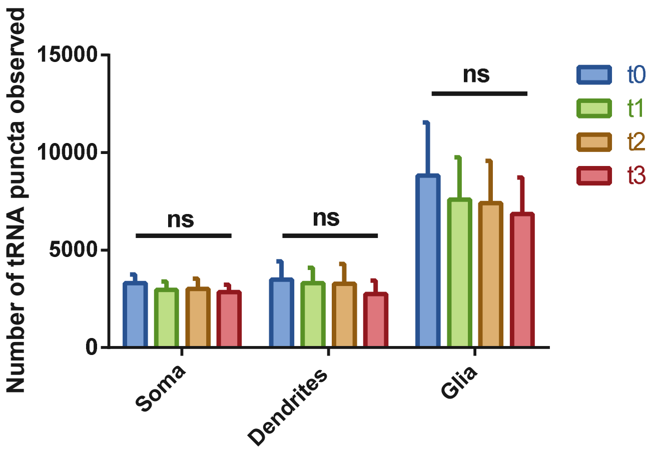

Figure 2 – figure supplement 2. Individual histograms of mean velocities of tRNA puncta in soma, dendrites and glia for each time point

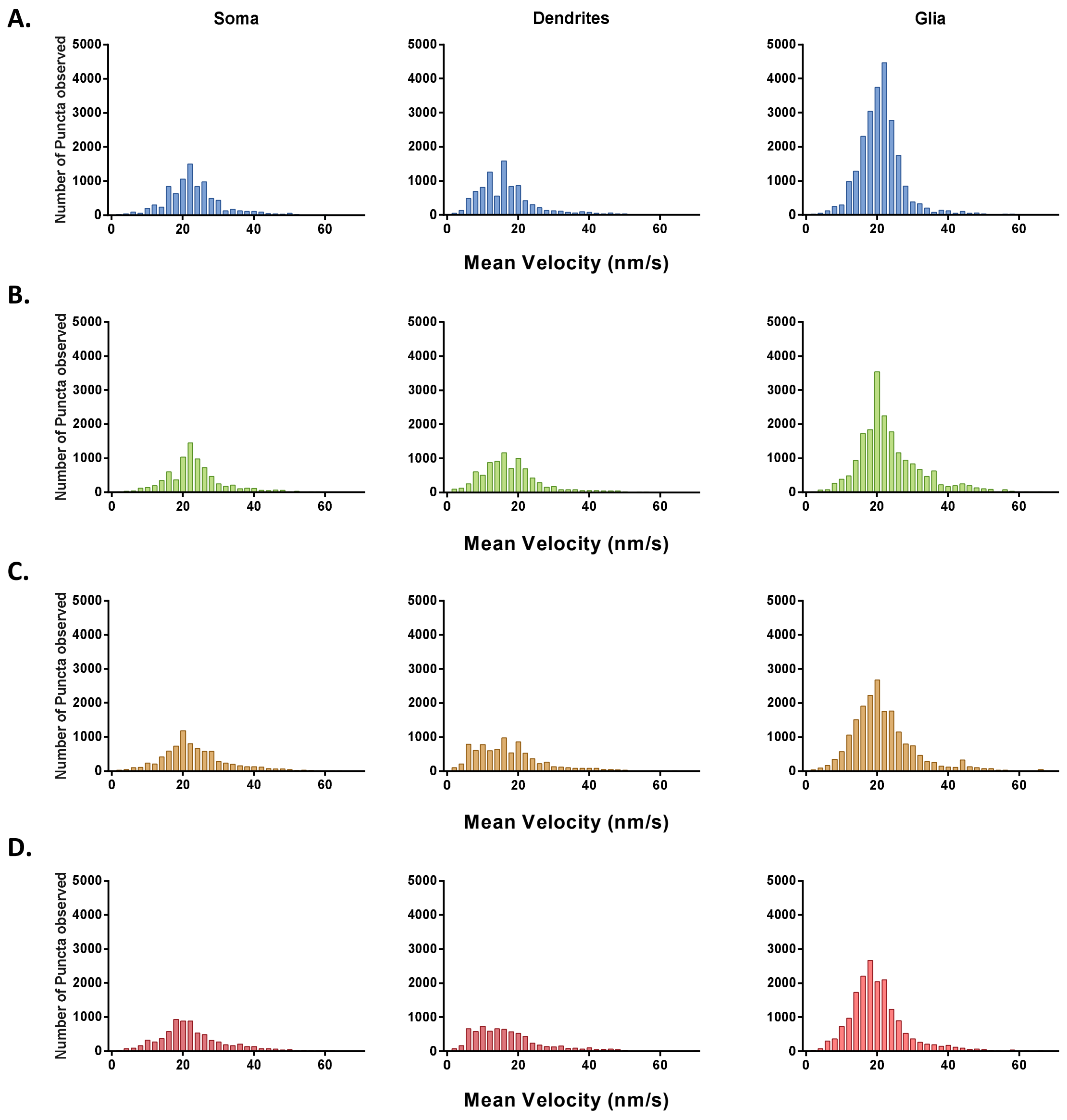

Figure 4 – figure supplement 1. Determination of the spatial resolution of dSTORM

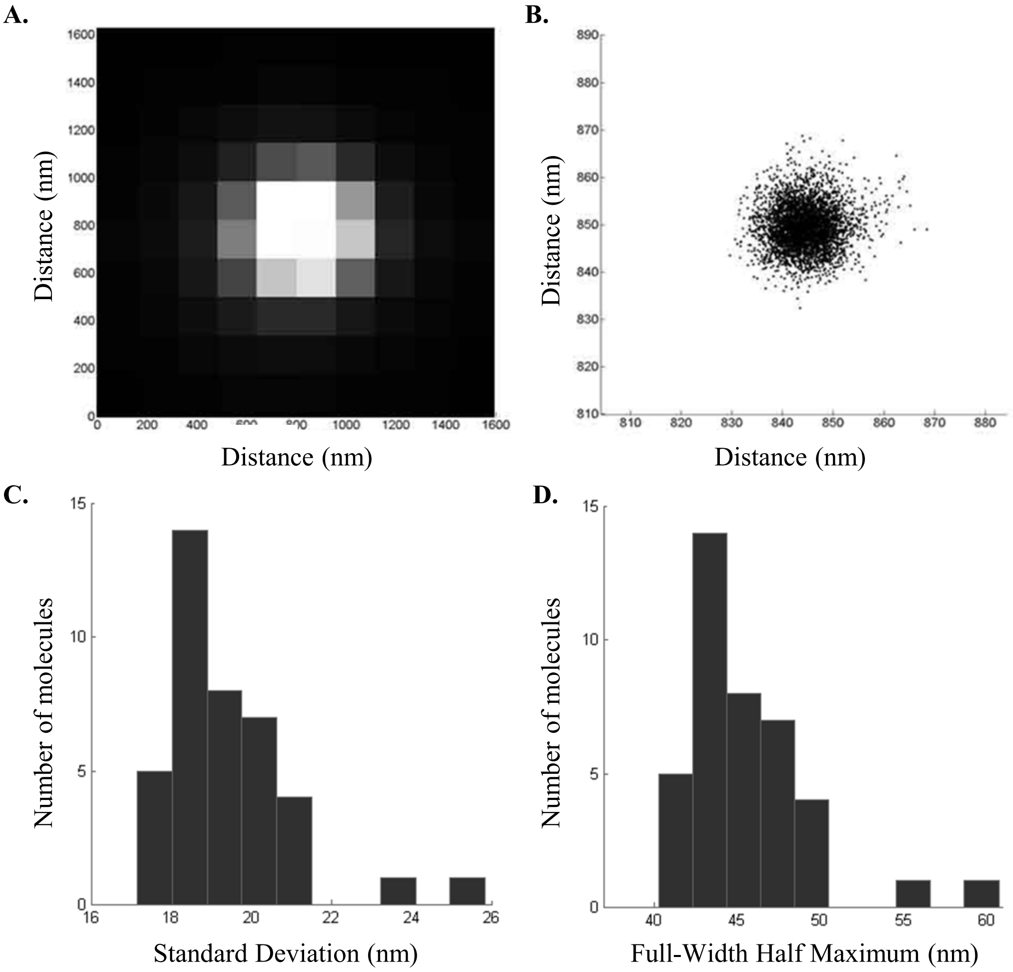

Figure 5 – figure supplement 1. Illustration of a Microfluidic chamber

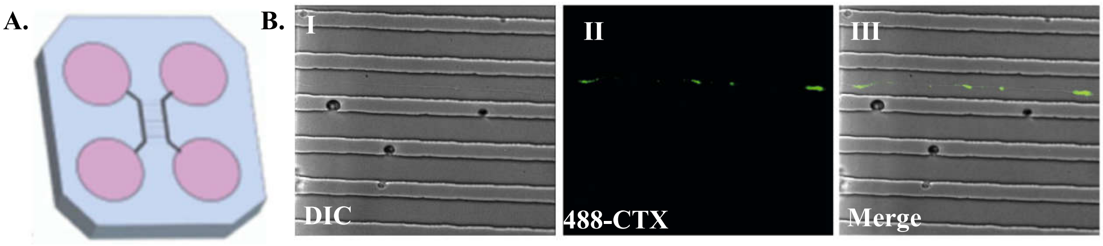

Figure 6 – figure supplement 1. Biphasic pattern of mRNA translation up-regulation is apparent in both neuronal somata and dendrites separately

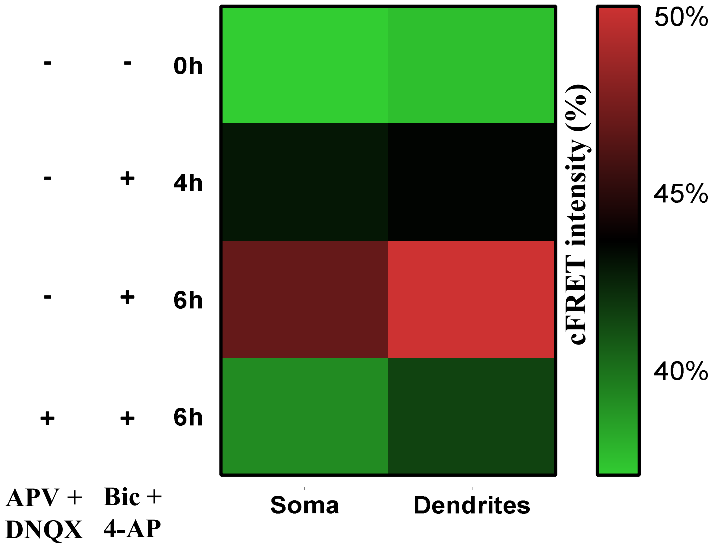

Figure 6 – figure supplement 2. mRNA translation up-regulation is inhibited by GluR antagonists only at 6h timepoint, but not at 2 and 4 hours.

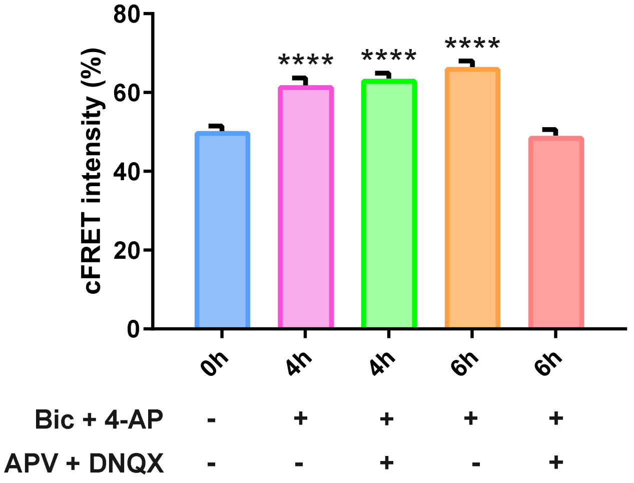

Figure 6 – figure supplement 3. Immunocytochemical SUnSET shows mRNA translation up-regulation following cLTP

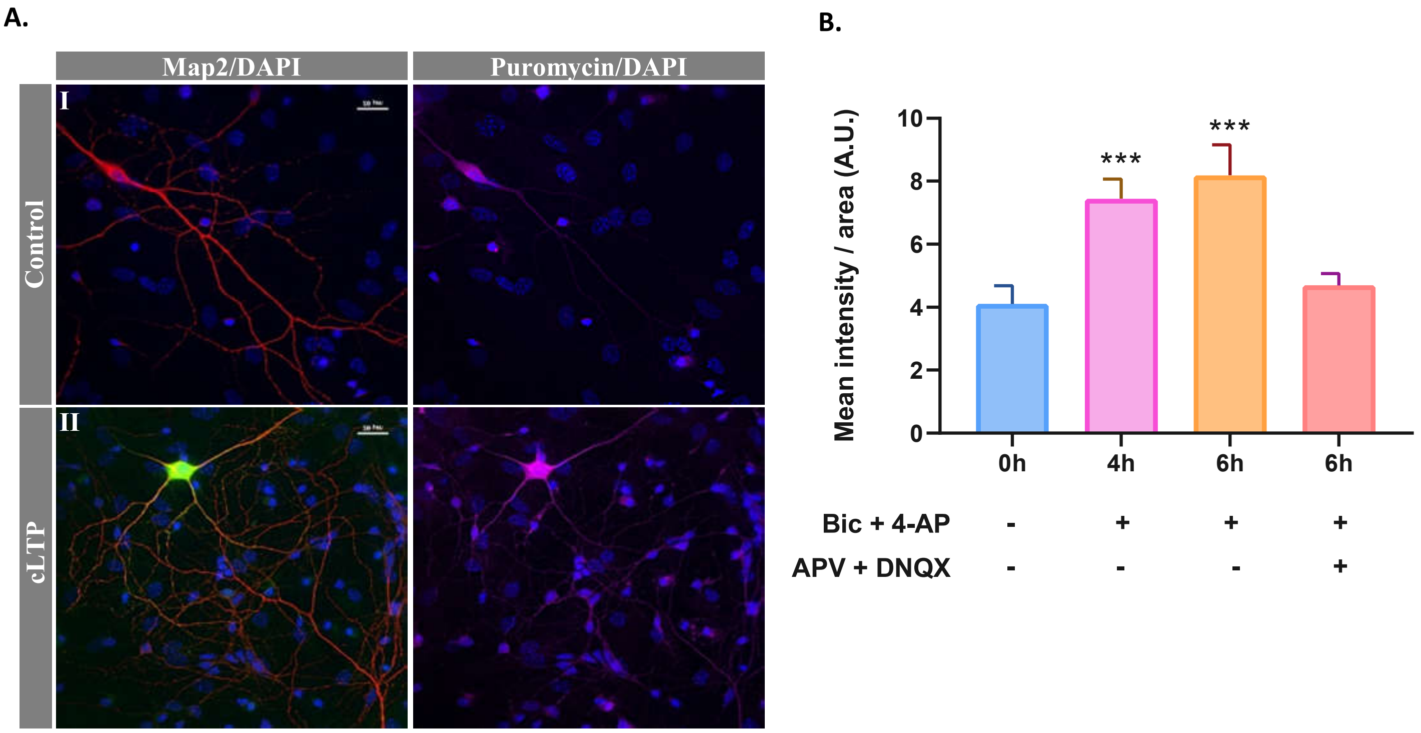

Supplement: gkaa042_Supplemental_Files [file gkaa042_supplemental_files.zip › Figure supplements 041219.pdf]
